# Evaluating the effect of massage based on slow stroke back massage on the anxiety of candidates for cataract surgery

**Published:** 2019

**Authors:** Reza Mohammadpourhodki, Mohammad Sadegh Sargolzaei, Mohammad Hasan Basirinezhad

**Affiliations:** * Nursing and Midwifery School, Shahroud University of Medical Sciences, Shahroud, Iran; ** BSc, Student of Nursing, Student Research Committee, Nursing and Midwifery School, Zabol University of Medical Sciences, Zabol, Iran.; *** MSc, Department of Epidemiology and Biostatistics, School of Public Health, Tehran University of Medical Sciences, Tehran, Iran

**Keywords:** slow-stroke-back massage, cataract, anxiety

## Abstract

**Purpose:** Preoperative anxiety over cataract surgery affects a large number of patients. This study aims to evaluate the effect of slow-stroke back massage on the anxiety of candidates for cataract surgery.

**Design:** Quasi-experimental study.

**Methods:** This study was a quasi-experimental study, which involved 60 candidates for cataract surgery referred to Amiralmomenin Hospital, Zabol in Iran between August 1, 2015, and March 30, 2016. These patients were randomly divided into two groups of slow-stroke back massage group (n=30) and control group (n=30). After obtaining an informed consent, the anxiety levels were measured by the Spielberger state trait anxiety questionnaire in the slow-stroke back massage group and the control group on the morning of the surgery before and immediately after the massage. SPSS software version 22 was used for data analysis. Independent t-test and chi-square test were used to compare the data.

**Findings:** According to the results, there was a significant difference between the anxiety levels of the patients in the intervention group before and after the massage (p < 0.001).

**Conclusions:** Based on the results of this study, Slow-stroke-back massage, which is a low-cost and safe method, significantly reduces anxiety in patients who are candidates for cataract surgery.

## Introduction

Cataract prevalence in the age group of 65-75 years is 50%, and reaches 70% in those over 75 years old. One million cataract surgeries are performed annually in the United States [**1**], currently cataract surgery being the only treatment [**2**]. Surgical stress causes serious physiological reactions and psychological reactions such as anxiety and fear [**3**]. The surgical anxiety along with the changes in physiological practice such as hypertension and increased heart rate can endanger the health of a patient. High levels of anxiety increase the risk of death three times [**4**]. 

Current therapies to modify physiological variables due to anxiety mainly focus on drug interventions. Therefore, sedatives and anti-anxiety drugs are prescribed before the operation. However, most drugs have adverse effects. Also, many of the non-pharmacological practices that are used today pertain to the field of complementary therapies. Research has shown that a variety of complementary medicines can affect anxiety prior to surgery [**4**]. Massage therapy is one of the most popular alternative and complementary therapies used in nursing, that is easy to implement, safe, non-invasive and relatively cheap [**5**]. In the case of massage, the slow-stroke back massage (SSBM) is reported to be a simple technique, inexpensive, rapid, and non-invasive and non-drug nursing intervention as well [**6**]. SSBM is a nursing intervention and a way of communication not by using words, but by touching the patient [**7**]. SSBM is, in fact, the gentle movement of the skin so that the hands slide over the skin and do not move the deep muscles [**8**]. SSBM is applied on the whole body. SSBM usually starts from the body’s posterior parts, the massage is slow, rhythmic and involves gentle movements of the hands on a patient’s back, at a speed of about 60 moves per minute, and it takes about 3 to 10 minutes. The movement used in this type of massage is a type of surface stroke that causes quite sensational effects and has very beneficial effects in patient relaxation [**9**]. Massage is one of the most important complementary therapies in nursing science that is considered by many nurses to be valuable and added to nursing skills, being able to provide comprehensive care [**10**]. 

Researchers have reported significant findings about the effectiveness of massage therapy on anxiety [**11**,**12**]. A study conducted by Baron and Faubert (2005) showed that anxiety decreased after massage therapy [**13**]. Albert et al. concluded that massage therapy was ineffective in reducing anxiety significantly [**14**]. Investigations showed that conducting surgery causes fear and anxiety in patients. Therefore, this study was designed and implemented to determine the effect of SSBM on the anxiety of patients undergoing cataract surgery.

## Methods 

The present study is a quasi-experimental research that involved 60 candidates for cataract surgery referred to Amiralmomenin Hospital, Zabol, in Iran, between August 2015, and March 2016.

The patients were randomly allocated into two groups; including intervention group (30 subjects) and control group (30 subjects). Matching in the groups was performed based on age, gender, marital status, employment status, and education level (**Table 1**).

**Table 1:** Demographic profile of patientsț two groups of Intervention and Control

Variable	Group	Intervention Number (%) or Mean (Standard deviation	Control Number (%) or Mean (Standard deviation	P-value
Age	41-50	8(26.7)	12(40)	†.206
	51-60	22(73.3)	18(60)	
Sex	Male	14(46.7)	18(60.0)	†.301
	Female	16(53.3)	12(40.0)	
Marital status	Married	28(93.3)	26(86.7)	†.335
	Single	2(6.7)	4 (13.3)	
Employment status	Employee	9 (30)	8 (26.7)	‡.246
	Self-employed	18 (60)	22 (73.3)	
	Housekeeper	3 (10)	0 (0)	
Location	City	29(96.7)	28(93.3)	† .554
	Village	1(3.3)	2(6.7)	
Level of Education	Illiterate	11(36.7)	16(53.3)	†.078
	Primary	8(26.7)	10(33.3)	
	Diploma	10(33.3)	2(6.7)	
	higher diploma	1(3.3)	2(6.7)	
* Independent t-test, † Chi-square test, ‡ Fisher’s Exact test				

After obtaining an informed consent for the participation in the research from the patients, they were considered as the sample of research. The inclusion criteria for the study were: referral for surgical treatment, medical diagnosis of cataract according to medical records, being at least 18 years old, not having known anxiety, not using relaxing and anti-anxiety medications the night before surgery, willingness to participate in the present study, having full vigilance, acceptable listening and speaking abilities to answer questions, and severity of anxiety above 20, based on the Spielberger state-trait anxiety questionnaire.

The exclusion criteria were death of patient during the study and withdrawal from the study.

Determination of sample size was based on the mean difference between intervention and control groups as suggested by in Jalalodini et al. [**11**]. The information-gathering tool consisted of two parts. The first part of the demographic information demographic questionnaire, which included questions about the participants’ demographic information such as age, sex, marital status, and educational level. The second part of Spielberger state trait anxiety questionnaire contained 20 questions of four answers with very low, low, high and very high options, and was completed through interviews with patients. The Spielberger state trait anxiety questionnaire had global validity and reliability. According to Mariam’s report for the mentioned test validity, the mean anxiety of normal and standard community in all age brackets was compared at 5% and 1%, to achieve a meaningful result, indicating the validity of anxiety measurement. The scientific reliability was also verified by α-Cronbach formula, which was 0.9452 in the normal community and 0.9418 in the standard community [**15**]. In addition, its reliability and validity in the Iranian society cardiac patients was confirmed via the study by Akbarzade et al. [**16**]. The questionnaire was made up of 20 multiple questions, with the options of “very little, little, a lot, and very much”. This questionnaire’s minimum score was 20 and the maximum was 80. In this research, the score 20–39 indicated mild anxiety, 40-59 indicated average anxiety, and 60-80 indicated intense anxiety [**17**]. 

The procedure of the study was such that the subjects were randomly divided into two groups of SSBM and control, then the demographic questionnaire was answered and the anxiety levels were measured by the Spielberger state-trait anxiety questionnaire in the SSBM and control groups on the morning of surgery before and after 15 minutes of the massage. Control group received routine intervention.

The slow-stroke-back massage procedures and program duration were explained to the test group comprising 30 participants and the massage was performed for the patients undergoing cataract surgery 30 minutes before the surgery by the researcher in a sitting position for each massage session. The duration of each massage session was 15 minutes.

The SSBM steps were the following: (1) the purpose and duration of massage were explained to the patient; (2) The privacy and security of the patients were observed. There were only the patient and the researcher in the massage room; (3) a participant would sit on the massage chair and lean his/ her head on the pillow; (4) before starting the massage, the searcher warmed up her hands by rubbing them together. To prevent damage to the patient’s skin, Vaseline was applied to the skin to lubricate the rubbing surface; (5) small circular strokes with thumbs on the neck (20 strokes in 30 seconds) were initiated; (6) surface stroke from the base of the skull to the sacral region using the palm of one hand and repeating the action on the other side of the spine using the palm of the other hand, while the first hand would move toward the base of the skull (60 strokes in 120 seconds); (7) hand strokes along the shoulder blades using the thumb (20 strokes in 30 seconds) followed; (8) hand strokes using the thumb on either side of the spine from shoulder to waist (10 strokes in 30 seconds) were next; (9) next came sweeping strokes from the neck area to the sacrum area using the palms of both hands (40 strokes in 90 seconds); (7) [**10**] steps 5 to 9 were repeated, and at the end of massage, the Vaseline was removed, and the surface of the participant’s skin was cleaned. The researcher has massage therapy certification from an accredited agency. The researcher carried out the massages alone, in a quiet place.

After 15 minutes of the massage session, the anxiety severity scale was completed by both groups. Control group received routine intervention.

An informed consent was obtained, and the research was approved with code zbmu.1.REC.1396.4 by ethics committee of Zabol University of Medical Sciences. In this study, the full description of the processes and the importance of the study were explained to the patients who had volunteered and were selected. All the assessments were non-invasive.

Statistical analysis of results was conducted using the SPSS-22 software in two parts of descriptive and inferential statistics. Descriptive statistics was used to describe data frequency: independent t-test and chi-square test were used to compare the data, and two-way variance analysis was used for hypothesis of having different anxiety means scores in two groups with repeated measurements.

## Results

The findings of this research showed that the demographic profiles of the patients were identical in both groups—intervention and control groups. Most of the subjects were male, married, self-employed, and lived in a city (**Table 1**).

Independent t-test showed that the mean score of anxiety in the intervention and control groups did not show significant difference before the massage (0.0915) (see **Table 2**).

**Table 2:** Mean and standard deviation of Anxiety between two groups of intervention and control group before and after interventions core in

Groups		Mean (± SD)	t	P value
Pre-intervention	Intervention (n=30)	49.7 (±5.43)	1.71	0.095
	Control (n=30)	47.16 (±6.02)		
Post-intervention	Intervention (n=30)	45.16 (±3.89)	-0.23	0.816
	Control (n=30)	45.43 (±5.02)		
* Independent t-test				

Paired t-test was used to examine the anxiety score of the patients before and after the intervention in both groups. The results indicated that the anxiety score in the intervention group (SSBM) decreased significantly (0.001), but in the control group, the decrease in anxiety score was not significant (0.231). Additional information is presented in **Table 3**. 

**Table 3:** Mean and standard deviation of Anxiety score in two groups of intervention and control group before and after intervention

Groups		Mean (± SD)	t	P value
Intervention (n=30)	Pre-intervention	49.7 (±5.43)	3.722	0.001
	Post-intervention	45.16 (±3.89)		
Control (n=30)	Pre-intervention	47.16 (±6.02)	1.208	0.231
	Post-intervention	45.43 (±5.02)		
*Paired t-test				

We also ran an ANCOVA analysis in order to control the role of the possible confounders’ variables and, as expected, because the baseline characteristics were not different between the two groups, the results of this analysis showed no significant difference after controlling confounders (data not shown).

## Discussion

 The results revealed that before the intervention, the mean of anxiety score in the two groups did not show any significant difference. After SSBM, the level of anxiety in the intervention group decreased to a mild degree, indicating an effect of this intervention method in reducing the anxiety of patients. 

In line with this study, the results of Baer et al.’s study, which was aimed at examining the effect of massage on the severity of pain and anxiety in patients undergoing cardiac surgery, showed that massage can reduce preoperative anxiety [**18**]. In a research titled the “effect of hand massage on preoperative anxiety in ambulatory surgery patients” in America, Brad and colleagues (2013) showed that patients who received routine nursing care along with hand massage experienced less anxiety [**19**]. Gholami Motlagh studied the effect of two methods of Swedish massage on vital signs and anxiety. The findings of that study indicated the positive effects of massage on vital signs and anxiety [**20**]. The results of the three above-mentioned studies are consistent with our study. Razmjoo et al. (2011) examined foot massage on women’s pain and anxiety after undergoing elective cesarean section; the results showed a significant difference in pain severity, but massage did not affect anxiety [**21**]. The results of that research were not consistent with the present study. The reason for the difference in the results of their research with the results of the present study may be due to the specific culture of the research community and the type of surgery. In a study by Ghezeljeh et al. (2017), there was no significant correlation between the effect of massage on the anxiety and pain of patients with burns; thus, the results of that study were not consistent with the findings of our study, and this difference may be due to the method of work and the sample because patients with burns experience higher levels of pain and anxiety [**22**]. The results of a study by Baron and Faubert (2005) showed that anxiety decreased after intervention [**13**]. The results of the present study on anxiety are consistent with the findings of the studies by Haun et al. (2009) [**23**] and Dreyer et al. (2015) [**24**]. In this study, the SSBM was used. However, this screening test only reveals the anxiety of the subjects. To improve the assessment, we recommend that in the future, study assessment is done by interviews and that patient satisfaction with the procedure is assessed.

The limitation of this study pertained to the inability to control potential intervention variables such as the emotional, physical, cultural, and social conditions, which were beyond the researcher’s control.

## Conclusion

According to the results of the present research and other similar studies, the non-pharmacological soothing of anxiety such as massage is effective in reducing the anxiety of patients undergoing cataract surgery. In addition, since nurses play an important role in treating and relieving the anxiety of patients and spend more time with cataract surgery patients than the other people in the health team, this method should be taken into consideration. According to the results of this study, it is recommended that other groups should also be investigated in this regard.

**Acknowledgments**

We would like to thank all the Faculty of Nursing and Midwifery University of Medical Sciences of Zabol, Honorable officials and all hospital patients.

**Author contributions**

All the authors have accepted responsibility for the entire content of this submitted manuscript and approved submission.

**Research funding**

This paper is the result of a research project at the Zabol University of Medical Sciences, which is sponsored by the university and the support is appreciated.

**Employment or leadership**

None declared.

**Honorarium**

None declared.

**Competing interests**

The funding organization(s) played no role in the study design; in the collection analysis, and interpretation of data; in the writing of the report; or in the decision to submit the report for publication.
